# Omentectomy for apparent early-stage uterine serous carcinoma: a large retrospective cohort study

**DOI:** 10.3389/fonc.2025.1656875

**Published:** 2025-11-27

**Authors:** Ouling Zhang, Yuedong He, Ai Zheng, Yu Xu

**Affiliations:** 1 Department of Obstetrics and Gynecology, West China Second University Hospital, Sichuan University, Chengdu, China; 2 Key Laboratory of Birth Defects and Related Diseases of Women and Children (Sichuan University), Ministry of Education, Chengdu, China

**Keywords:** uterine serous carcinoma, omentectomy, overall survival, disease-free survival, endometrial cancer

## Abstract

**Objectives:**

This study aims to compare the effects of different omental assessment methods (omentectomy and omental biopsy) on the long-term prognosis of apparent early-stage uterine serous cancer (USC).

**Methods:**

A total of 255 women with clinical early-stage USC were included. They were divided into the omentectomy group and the omental biopsy group. The Kaplan-Meier method and the log-rank test were employed to estimate and compare overall survival and disease-free survival between groups. The Cox proportional hazards regression model was used to adjust for potential confounding factors.

**Results:**

When compared with undergoing omentectomy, women with apparent early-stage USC who underwent omental biopsy had a deteriorated 5-year OS (HR: 1.76, 95% CI: 1.07-3.52, *P*=0.009) and 5-year DFS (HR: 1.72, 95% CI: 1.15-2.86, *P*=0.012). After adjusting for confounding factors, omental biopsy was independently associated with worsening long-term prognosis in apparent early-stage USC (For DFS, aHR=1.78, 95% CI: 1.13-3.20, *P*=0.025; For OS, aHR=1.68, 95% CI: 1.11-2.75, *P*=0.041).

**Conclusions:**

For apparent early-stage USC, in terms of long-term survival outcomes, omentectomy is superior to omental biopsy.

## Introduction

Uterine serous carcinoma (USC) is a rare type of endometrial cancer, accounting for approximately 15% of endometrial cancers (1, 2). Although rare, about 40-50% of adequately staged cases do have extrauterine metastasis, accounting for more than 50% of recurrences and endometrial cancer-related deaths (1, 3, 4). The 5-year overall survival rate of patients with USC is poor, and the data reported in the literature range from 18% to 27% (5, 6). Therefore, to improve the overall survival outcomes of endometrial cancer, attention should be paid to treating patients with USC.

Like endometrioid cancer, the standard surgical staging for apparent early-stage USC includes total hysterectomy, bilateral salpingo-oophorectomy, and regional lymph node assessment (7–10). Minimally invasive surgery is the recommended surgical approach according to the guidelines (7–10). For pathological subtypes that are prone to extrauterine metastasis, omental assessment or peritoneal biopsies, or both, are recommended (7, 10). However, the recommendations vary among different guidelines regarding the omental evaluation for women with USC. According to the National Comprehensive Cancer Network (NCCN) Guideline for endometrial cancer, omental biopsy is recommended for women with USC (11). However, omentectomy, not omental biopsy, is mandatory for surgical staging for apparent early-stage USC according to the consensus of the European Society for Medical Oncology and European Society for Radiotherapy & Oncology and European Society of Gynaecological Oncology (ESMO-ESGO-ESTRO) (12).

A considerable number of studies have reported the incidence of omental metastasis in patients with USC (13–15). However, few have evaluated the long-term oncological survival differences of patients with USC after receiving different omental evaluations. To investigate the impact of different omental assessment methods (omentectomy versus omental biopsy) on the long-term survival outcomes of patients with USC, we designed and implemented this study.

## Methods

Based on a large-volume center (West China Second University Hospital, Sichuan University), this is a retrospective cohort study. The Institutional Review Board of West China Second University Hospital exempted the approval to conduct this study based on the fact that it did not involve patients’ private information and the retrospective design. We conducted this study by following the Declaration of Helsinki (16).

### Study cohort

We enrolled patients with USC who underwent consecutive management at the West China Second University Hospital between January 1, 2018, and December 31, 2023. A thorough review of the medical records of the included patients was performed. Their medical records were extracted by the International Classification of Diseases 9th and 10th Revisions searches.

The inclusion criteria of this study were as follows: 1) Be under 75 years old at diagnosis. 2) No clinical or imaging evidence (ultrasound, magnetic resonance imaging, or computed tomography) indicating that the disease is at an advanced stage. In this study, clinically or radiologically suspected metastases in the following sites were regarded as clinically advanced cases: the outer layer of the uterus, the oviduct, the ovary, the ligament of uterus, the vagina, the parametrium of the uterus, the pelvic and para-aortic lymph node, and organs beyond the pelvis. 3) With a pathological diagnosis of USC. In this study, based on the point of the Gynecologic Oncology Group Pathology Committee, only cases where the USC component in the tumor exceeds 50% were designated as USC (17). 4) Underwent adequate surgical staging, including total hysterectomy, bilateral salpingo-oophorectomy, pelvic and para-aortic lymphadenectomy, and omental assessment. 5) Underwent consecutive postoperative follow-up as recommended.

The exclusion criteria of this study were as follows: 1) Were lost to follow-up. 2) Had another malignancy at the same time. 3) Be in an immunosuppressive state, including the Acquired Immune Deficiency Syndrome, after organ transplantation, and primary immunodeficiency. 4) with incomplete data of interest.

### Data collection

In this study, the data of interest were as follows: the year of diagnosis, the patients’ age at diagnosis, the patients’ body mass index (BMI) at diagnosis, the American Society of Anesthesiologists (ASA) physical status score when the patients underwent operation, the diameter of the tumor, the result of peritoneal cytology, the status of lymphovascular space invasion (LVSI), the surgical approach, the protocol of postoperative adjuvant management (chemotherapy, radiation, or chemoradiotherapy), and the final surgical pathological stage of the disease. In this study, by reviewing the findings during surgery and the report of pathological examination, we would stage all the included cases again based on the International Federation of Gynecology and Obstetrics 2023 staging system for endometrial cancer.

### Outcomes of interest

In our study, all enrolled cases were followed up until January 1, 2025, or death. We collected data on survival outcomes as follows: whether alive, whether the disease recurs, the time of recurrence, and the time of death.

In this study, the primary outcomes of interest were overall survival (OS) and disease-free survival (DFS). The former is defined as the duration from the beginning of therapy for USC to the date of death from any cause or the last follow-up, and the latter is defined as the duration from the beginning of therapy for USC to the date of disease recurrence, death from any cause, or the last follow-up. The prognostic factors for the survival of USC were also the outcomes of interest.

### Statistical analysis

In this study, according to the type of data, the Student’s *t*-test, the Fisher’s exact test, the χ2 test, and the Wilcoxon rank-sum test were performed to analyze the baseline characteristics and clinico-pathological variables. The DFS and OS survival curves were constructed using the Kaplan-Meier method, and the comparison of DFS and OS between cohorts was performed by using the log-rank test. Using the Cox proportional hazard regression test, the prognostic effect of clinicopathological variables on PFS and OS was estimated and presented as hazard ratios (HRs) and 95% confidence intervals (CIs). All tests were two-tailed, and we considered results of *P*<0.05 as statistically significant.

## Results

### Cohort characteristics

In total, 255 women with apparent early-stage USC were included in our study after careful review of their medical records. Among them, 84 patients (32.9%) underwent omentectomy (omentectomy group), and 171 (67.1%) patients underwent omental biopsy (omental biopsy group). The baseline characteristics, clinicopathologic characteristics, and treatment variables are presented in **Table 1**. Overall, all included patients underwent long-term follow-up, with a median follow-up of 40 months (1 to 107). There were no statistically significant differences in age at diagnosis (*P*=0.940), BMI at diagnosis (*P*=0.099), ASA physical score at operation (*P*=0.502), the diameter of the primary tumor (*P*=0.732), the result of peritoneal cytology (*P*=1.000), the report of LVSI (*P*=0.871), the FIGO stage (*P*=0.442), and surgical approach (*P*=0.560) between the two groups, except for whether they underwent postoperative therapy (*P*<0.001). For the entire study cohort, after surgical staging, 24.7% of cases initially classified as being in the clinical early stage were found to be in advanced stages.

**Table 1:** Clinicopathologic characteristics of the study cohort.

	Overall (N=255)	Omentectomy group (N=84)	Omental biopsy group (N=171)	*P*
Year of diagnosis				0.066
2018-2020	85 (33.3%)	35 (41.7%)	50 (29.2%)	
2021-2023	170 (66.7%)	49 (58.3%)	121 (70.8%)	
Age at diagnosis				0.940
Mean (SD)	64.2 (6.02)	64.2 (6.20)	64.2 (5.95)	
Median [Min, Max]	64.0 [39.0, 74.0]	64.0 [43.0, 74.0]	64.0 [39.0, 74.0]	
Body mass index				0.099
<25 kg/m^2^	215 (84.3%)	66 (78.6%)	149 (87.1%)	
≥25 kg/m^2^	40 (15.7%)	18 (21.4%)	22 (12.9%)	
ASA score				0.502
I/II	150 (58.8%)	52 (61.9%)	98 (57.3%)	
III/IV	105 (41.2%)	32 (38.1%)	73 (42.7%)	
Follow-up duration				0.724
Mean (SD)	42.6 (24.9)	43.5 (28.4)	42.2 (23.0)	
Median [Min, Max]	40.0 [1.00, 107]	40.5 [1.00, 107]	40.0 [2.00, 103]	
Tumor size				0.732
<4cm	208 (81.6%)	70 (83.3%)	138 (80.7%)	
≥4cm	47 (18.4%)	14 (16.7%)	33 (19.3%)	
Peritoneal cytology				1.000
Negative	178 (69.8%)	59 (70.2%)	119 (69.6%)	
Positive	77 (30.2%)	25 (29.8%)	52 (30.4%)	
LVSI				0.871
Negative	201 (78.8%)	67 (79.8%)	134 (78.4%)	
Positive	54 (21.2%)	17 (20.2%)	37 (21.6%)	
FIGO stage (2023)				0.442
I/II	192 (75.3%)	66 (78.6%)	126 (73.7%)	
III/IV	63 (24.7%)	18 (21.4%)	45 (26.3%)	
Surgical approach				0.560
MIS	182 (71.4%)	58 (69.0%)	124 (72.5%)	
Open	73 (28.6%)	26 (31.0%)	47 (27.5%)	
Postoperative therapy				<0.001
No	97 (38.0%)	9 (10.7%)	88 (51.5%)	
Yes	158 (62.0%)	75 (89.3%)	83 (48.5%)	

SD, standard deviation; ASA, American Society of Anesthesiologists; LVSI, lymphovascular space invasion; FIGO, the International Federation of Gynecology and Obstetrics; MIS, minimally invasive surgery.

### Prevalence of omental metastases in apparent early-stage USC

Overall, 47 (18.4%) out of 255 included patients had omental metastases confirmed by postoperative pathological examination, all presented as occult disease. For the omentectomy group, the omental metastases rate was 26.19%. while in the omental biopsy group, 25(14.62%) out of 171 women had occult omental metastases.

### Survival outcomes

**Figure 1:**
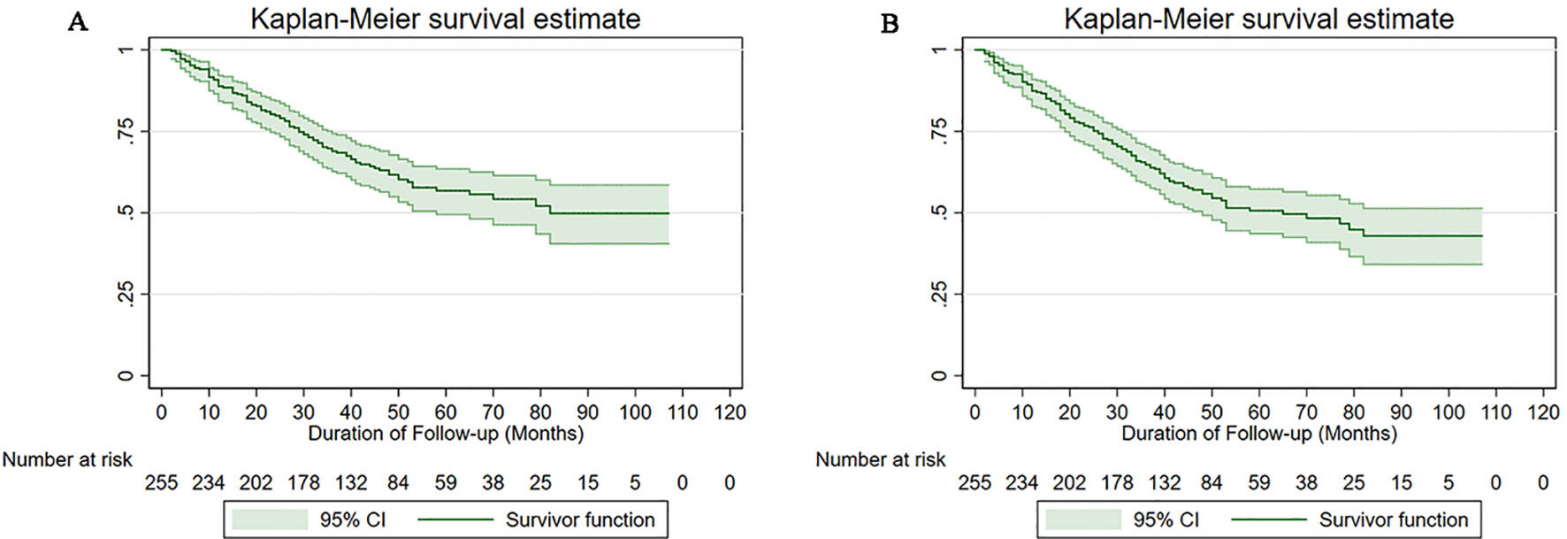
Survival curves of the study cohort **(A)** for overall survival, **(B)** for disease-free survival.

At the follow-up endpoint of this study, there were 24 (28.6%) and 75 (43.9%) all-cause deaths in the omentectomy group and the omental biopsy group, respectively. **Figure 1** presents the Kaplan-Meier survival curves of OS (**Figure 1A**) and DFS (**Figure 1B**) for all the included cases in our study. Overall, for the study cohort, the 5-year DFS and 5-year OS were 56.79% (95% CI: 49.47%-63.45%) and 50.64% (95% CI: 43.59%-57.25%), respectively.

**Figure 2:**
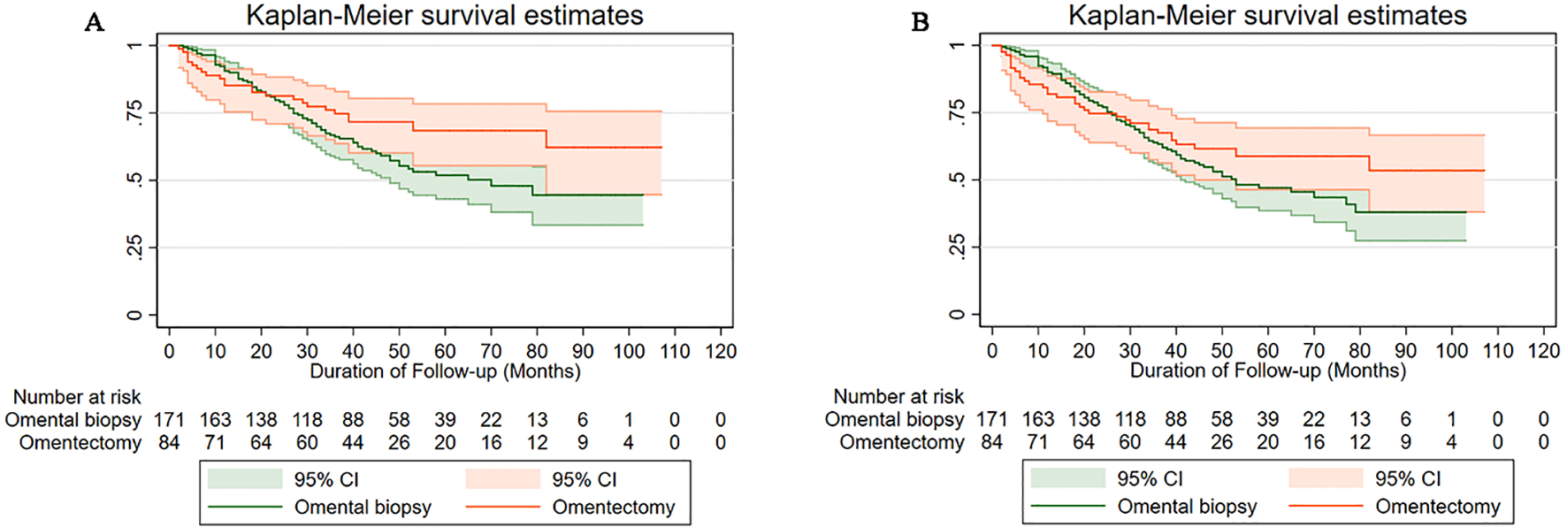
Survival curves of the study cohort by type of omental assessment **(A)** for overall survival, **(B)** for disease-free survival.

The 5-year DFS rate by Kaplan-Meier method was 58.82% (95% CI: 46.38%-69.32%) in the omentectomy group and 47.06% (95% CI: 38.56%-55.10%) in the omental biopsy group. For women with apparent early-stage USC, when compared to undergoing omentectomy, undergoing omental biopsy was associated with a deteriorated 5-year DFS (HR: 1.72, 95% CI: 1.15-2.86, *P*=0.012). The 5-year OS rate in the omentectomy group and the omental biopsy group were 68.44% (95% CI: 55.42%-78.37%) and 51.89% (95% CI: 43.06%-60.00%), respectively. The log-rank test indicated that when compared with undergoing omentectomy, women with apparent early-stage USC who underwent omental biopsy had a deteriorated 5-year OS (HR: 1.76, 95% CI: 1.07-3.52, *P*=0.009). **Figure 2** shows the Kaplan-Meier survival curves of OS (**Figure 2A**) and DFS (**Figure 2B**) for the omentectomy group and the omental biopsy group.

### Univariate and multivariate analysis of the prognostic factors for apparent early-stage USC

To identify the association between the variables (clinical, pathological, and treatment) and the long-term survival outcomes of patients with apparent early-stage USC, a univariate analysis using the Kaplan-Meier method and the log-rank test was performed. **Table 2** shows the results of the univariate analysis. As **Table 2** presents, for apparent early-stage USC, high ASA physical score (HR=1.87, 95% CI: 1.28-2.72, *P*=0.001), larger primary tumor size (HR=1.75, 95% CI: 1.06-2.89, *P*=0.009), positive LVSI (HR=1.93, 95% CI: 1.20-3.10, *P*=0.008), advanced FIGO stages (HR=3.13, 95% CI: 1.97-4.96, *P*=0.000), and no postoperative therapy (HR=3.11, 95% CI: 2.12-4.57, *P*=0.000) were associated with worse DFS. In terms of the OS of apparent early-stage USC, the aforementioned factors also have the same impact, as follows: ASA physical score (III/IV versus I/II: HR=1.66, 95% CI: 1.10-2.50, *P*=0.011), tumor size (≥4cm versus <4cm: HR=2.02, 95% CI: 1.16-3.50, *P*=0.002), LVSI (positive versus negative: HR=1.84, 95% CI: 1.09-3.10, *P*=0.030), FIGO stages (III/IV versus I/II: HR=3.38, 95% CI: 2.04-5.61, *P*=0.000), and postoperative therapy (no versus yes: HR=4.00, 95% CI: 2.62-6.09, *P*=0.009).

**Table 2:** Univariate analysis of prognostic factors for DFS and OS in apparent early-stage uterine serous cancer.

	DFS			OS		
	HR	95% CI	*P*	HR	95% CI	*P*
Age at diagnosis						
<65 years	1			1		
≥65years	1.17	0.82-1.68	0.381	1.17	0.79-1.73	0.438
Body mass index						
<25 kg/m^2^	1			1		
≥25 kg/m^2^	1.19	0.72-1.96	0.467	1.07	0.62-1.85	0.799
ASA score						
I/II	1			1		
III/IV	1.87	1.28-2.72	0.001	1.66	1.10-2.50	0.011
Tumor size						
<4cm	1			1		
≥4cm	1.75	1.06-2.89	0.009	2.02	1.16-3.50	0.002
Peritoneal cytology						
Negative	1			1		
Positive	1.17	0.79-1.75	0.434	1.03	0.66-1.59	0.905
LVSI						
Negative	1			1		
Positive	1.93	1.20-3.10	0.008	1.84	1.09-3.10	0.03
FIGO (2023)						
I/II	1			1		
III/IV	3.13	1.97-4.96	0.000	3.38	2.04-5.61	0.000
Surgical approach						
MIS	1			1		
Open	0.88	0.59-1.32	0.555	0.85	0.55-1.31	0.476
Postoperative therapy						
Yes	1			1		
No	3.11	2.12-4.57	0.000	4.00	2.62-6.09	0.000
Omental assessment						
Omentectomy	1			1		
Omental biopsy	1.72	1.15-2.86	0.012	1.76	1.07-3.52	0.009

DFS, disease-free survival; OS, overall survival; HR, hazard ratio; CI, confidence interval; ASA, American Society of Anesthesiologists; LVSI, lymphovascular space invasion; FIGO, the International Federation of Gynecology and Obstetrics; MIS, minimally invasive surgery.

The Cox proportional hazards regression analysis was performed to adjust for the unbalanced confounding factors between the two groups. The following factors were included in the Cox proportional hazards regression analysis model: ASA score, tumor size, LVSI, FIGO stage, postoperative therapy, and the approach of omental assessment. The Cox proportional hazards regression analysis shows that for apparent early-stage USC, the evaluation method of omentum is independently correlated with the long-term prognosis of patients (omental biopsy versus omentectomy: For DFS, aHR=1.78, 95% CI: 1.13-3.20, *P*=0.025; For OS, aHR=1.68, 95% CI: 1.11-2.75, *P*=0.041). **Table 3** presents the results of the Cox proportional hazards regression analysis.

**Table 3:** Multivariate Cox proportional hazards analyses for predictors of DFS and OS in apparent early-stage uterine serous cancer, with selection of covariates using stepwise forward selection (P < 0.2).

	DFS			OS		
	aHR	95% CI	*P*	aHR	95% CI	*P*
ASA score						
I/II	1			1		
III/IV	1.88	1.04-2.98	0.008	1.67	1.08-2.39	0.014
Tumor size						
<4cm	1			1		
≥4cm	1.04	0.65-1.68	0.861	1.18	0.89-1.49	0.667
LVSI						
Negative	1			1		
Positive	1.94	1.05-3.17	0.036	2.03	1.17-3.80	0.010
FIGO (2023)						
I/II	1			1		
III/IV	2.74	1.15-3.61	0.008	2.86	1.08-4.63	0.022
Postoperative therapy						
Yes	1			1		
No	3.08	2.04-4.65	0.000	3.75	2.36-5.95	0.000
Omental assessment						
Omentectomy	1			1		
Omental biopsy	1.78	1.13-3.20	0.025	1.68	1.11-2.75	0.041

DFS, disease-free survival; OS, overall survival; aHR, adjusted hazard ratio; CI, confidence interval; ASA, American Society of Anesthesiologists; LVSI, lymphovascular space invasion; FIGO, the International Federation of Gynecology and Obstetrics.

## Discussion

In this study, we found that in apparent early-stage USC, the risk of occult omental metastases is high, 18.4% in the entire study cohort. We also found that the apparent early-stage USC patients who underwent omentectomy had a better long-term prognosis compared to those who only underwent omental biopsy. Based on these findings, omentectomy rather than omental biopsy should be an essential part of surgical staging for apparent early-stage USC.

Although originating from the endometrium, in terms of biological behavior, USC is more similar to high-grade serous adenocarcinoma of the ovary rather than endometrioid adenocarcinoma (18–20). Considering its invasive and aggressive growth behavior, a gynecological oncologist often employs more comprehensive surgical staging to manage this malignancy (18–20). The theoretical basis behind this clinical procedure is the inclination of USC to shed tumor cells and metastasize widely to sites outside the uterus, including omentum, appendix, pelvic peritoneum, etc. However, there is still debate about whether omentectomy should be an essential part of comprehensive surgical staging for apparent early-stage USC. Omental biopsy is recommended by the National Comprehensive Cancer Network guidelines, while the ESMO-ESGO-ESTRO guideline recommends omentectomy as a regular part of surgical staging in apparent early-stage USC (21).

In USC, the prevalence of omental metastases varies from study to study, ranging from 10% to 18% (13, 22–24). A meta-analysis involving 1012 cases of USC found that the overall omental metastases rate, the gross omental metastases rate, and the occult omental metastases rate are 18%, 6%, and 10%, respectively (13). Studies reported that even in the clinical early-stage USC, about one-third of cases have abdominal metastasis of cancer, including the omentum (25, 26). Therefore, the risk of microscopic omental metastases in patients with USC should not be underestimated. One study by Kaban et al. found that in nonendometrioid-type endometrial malignancy, nearly half (44.1%) of the omental metastases are microscopic (24). They also found that the result of the omental assessment by the surgeon’s visual is not sensitive (24). All of these indicate that in USC, the visual assessment of the omentum is not sufficient for the identification of omental metastases.

Although the omentum has long been considered a disease defense mechanism within the abdominal cavity, for apparent early-stage USC, we prefer omentectomy over omental biopsy for the following reasons. First, the ability of the omentum to trap and inhibit tumor cells may not be sufficient to limit tumor metastasis in the abdominal cavity (27, 28). The adipose in the omentum also works as fuel to facilitate the growth and spread of tumor cells (29). Second, optimal debulking has a positive long-term prognostic effect on USC, just like it does on high-grade serous adenocarcinoma of the ovary. Patients with omental metastases are staged at an advanced stage. For these patients, omentectomy, on the one hand, can prevent residual effects of occult omental metastases, and on the other hand, can guide postoperative management. Third, a considerable proportion of cancer recurrence occurs in the omentum. One study conducted by Luz R reported that in USC, the second most common recurrence site is the omentum (27%), following closely behind the peritoneum (31%) (23). The last, with the development of surgical techniques, omentectomy is no longer a complex surgical procedure and rarely leads to serious complications (30).

For a rare subtype of endometrial cancer, our study enrolled a relatively large sample and underwent a long duration of follow-up. However, our study has several limitations. First, because of the retrospective design, there are some inherent biases, such as recall bias and referral bias. Second, our study is just a single-center study and can not well represent the Chinese population. Third, due to limited resources, the postoperative pathological examination report was not reviewed by another pathologist. However, considering the low prevalence of USC, our research has added some depth to the understanding of this topic.

In conclusion, for apparent early-stage USC, in terms of long-term survival outcomes, omentectomy is superior to omental biopsy.

## Data Availability

The raw data supporting the conclusions of this article will be made available by the authors, without undue reservation.
